# Higher Medical Legislation

**Published:** 1892-12

**Authors:** W. F. Westmoreland


### HIGHER MEDICAL LEGISLATION.

Ye honest physicians who have so long listened with credulity to the whispers of fancy, and pursued with eagerness the phantoms of hope, can expect this legislatnre instead of age to perform the promise of higher medical legislation, and that the deficiences of the present day will be supplied during this session.

The bill just passed by the senate and transmitted to the house does and should have the endorsement and fullest sympathy of the medical profession of Georgia. We as a journal heartily approve it, and sincerely hope it will pass the house by the same decided vote it did the senate, and we are sure that if the different members of the legislature could look at it from the standpoint that it means protection to themselves and families, through the fact that in the future every man who expects to practice medicine in this State will not only have to attend an additional year at a medical college, but also come up before a conscientious examining board before he can qualify as a physician—a board appointed by the governor; and a particularly strong point in reference to this board is the fact that no member of the faculty of any medical college can be on the board, and that it puts the licensing power of their students to practice into other hands. Certainly all the medcal colleges of this State which have been so clamorous through the medical journals for higher medical education, should do all in their power to assist the passage of this bill.

For I am sure that there is no teacher in any school, which only requires two terms of lectures, that does not feel, if he is at all conscientious, that these men his school graduates, would be infinitely better *qualified* to practice medicine if they could be kept three years.

The bill is in every respect a good, strong, conservative one, and will meet a long felt want—that of protecting the people from illegal and unqualified practitioners, and in the future a physician will come to you with the endorsement of his State as well as his college, and it gives the greatest pleasure to see our State arraying herself in line with the surrounding States as being in favor of higher medical legislation.

W. F. WESTMORELAND.

